# ARE-mediated decay controls gene expression and cellular metabolism upon oxygen variations

**DOI:** 10.1038/s41598-018-23551-8

**Published:** 2018-03-26

**Authors:** Bérengère de Toeuf, Romuald Soin, Abdelkarim Nazih, Marija Dragojevic, Dukas Jurėnas, Nadège Delacourt, Long Vo Ngoc, Abel Garcia-Pino, Véronique Kruys, Cyril Gueydan

**Affiliations:** 1 Laboratoire de Biologie Moléculaire du Gène, Faculté des Sciences, Université libre de Bruxelles (ULB), 12 rue des Profs. Jeener et Brachet, 6041 Gosselies, Belgium; 2 Laboratoire de Microbiologie Moléculaire et Cellulaire, Faculté des Sciences, Université libre de Bruxelles (ULB), 12 rue des Profs. Jeener et Brachet, 6041 Gosselies, Belgium; 3 Present Address: Section of Molecular Biology, University of California at San Diego, La Jolla, California 92093 USA

## Abstract

Hypoxia triggers profound modifications of cellular transcriptional programs. Upon reoxygenation, cells return to a normoxic gene expression pattern and mRNA produced during the hypoxic phase are degraded. TIS11 proteins control deadenylation and decay of transcripts containing AU-rich elements (AREs). We observed that the level of dTIS11 is decreased in hypoxic S2 *Drosophila* cells and returns to normal level upon reoxygenation. Bioinformatic analyses using the ARE-assessing algorithm AREScore show that the hypoxic S2 transcriptome is enriched in ARE-containing transcripts and that this trend is conserved in human myeloid cells. Moreover, an efficient down-regulation of *Drosophila* ARE-containing transcripts during hypoxia/normoxia transition requires *dtis11* expression. Several of these genes encode proteins with metabolic functions. Here, we show that *ImpL3* coding for Lactate Dehydrogenase in *Drosophila*, is regulated by ARE-mediated decay (AMD) with dTIS11 contributing to *ImpL3* rapid down-regulation upon return to normal oxygen levels after hypoxia. More generally, we observed that *dtis11* expression contributes to cell metabolic and proliferative recovery upon reoxygenation. Altogether, our data demonstrate that AMD plays an important role in the control of gene expression upon variation in oxygen concentration and contributes to optimal metabolic adaptation to oxygen variations.

## Introduction

ARE-mediated decay (AMD) defines a mechanism leading to the rapid degradation of messenger RNAs (mRNA) due to the presence of AU-rich elements (AREs) in their 3′ untranslated regions (3′UTRs). Since their discovery as major regulatory elements controlling inflammation^1–4^, AREs have been found to play major roles in several fundamental biological processes such as growth, differentiation and apoptosis^5^. One common feature of ARE-containing genes is their transient expression profile, ARE enrichment determining the temporal profile of gene expression^6^. Although the question of which consensus sequence constitutes a functional ARE has been a long-debated topic^7^, analysis of mammalian transcript 3′UTRs based on rather restrictive consensus indicates that these elements are the most common *cis*-acting elements and are found in approximately 11% of the total gene number in human ENSEMBL database^8^.

AREs are bound by a large variety of RNA-binding proteins which affect negatively or positively the expression of their target ARE-containing mRNA (for reviews see: refs^8,9^). For example, proteins of the TIS11/TTP and AUF1 families most frequently stimulate mRNA rapid degradation^10,11^ while proteins of the Hu family act as mRNA stabilizers^12^. Other ARE-binding proteins (ARE-BPs) such as TIAR and TIA-1 inhibit mRNA translation^13,14^. Therefore, the outcome of ARE-mediated gene regulation is determined by the availability of ARE-BPs and their affinity for the ARE present in the mRNA 3′UTR^15–17^.

AMD emerged early in the evolution of eukaryotes as ARE are found in transcripts from fungi^18^. Moreover, the TIS11/TTP proteins which are composed by a CCCH tandem zinc finger common to all members are also highly conserved in eukaryotes (refs^19,20^ for review). While AMD controls the stability of a wide range of transcripts in mammals, it regulates different restricted sets of genes in unicellular organisms. In *Saccharomyces cerevisiae*, AMD mainly controls genes involved in iron metabolism. In contrast, AMD targets genes involved in cell-cell interactions in *Schizosaccharomyces pombe* and other unrelated transcripts in *Candida albicans*. These observations reveal that despite the conservation of AMD, species have evolved their specific set of targets to meet their specific physiological requirements^18^.

ARE are well represented regulatory motifs in invertebrates. A bioinformatic analysis of *D*. *melanogaster* genome predicts a widespread contribution of AREs to post-transcriptional regulation in this organism and indicates that these elements are highly conserved across *Drosophila* species^21^. Interestingly, a 3-fold enrichment of genes containing an ARE is found in *Drosophila* immune-induced genes, suggesting that AMD evolved early in evolution as an important regulatory mechanism of the immune response^21^. The functional role of ARE in *Drosophila* has been demonstrated *in vitro* and *in vivo* and degradation of ARE-containing mRNA is promoted by the binding of dTIS11, the sole member of the TIS11/TTP family in this organism^21,22^.

TIS11/TTP protein accumulation is tightly controlled by multiple regulatory mechanisms acting at the transcriptional, post-transcriptional and post-translational levels (see ref.^23^, for review). We recently described that *Drosophila* and mammalian TIS11/TTP proteins are short-lived due to rapid ubiquitin-independent degradation by the proteasome and that this mechanism is tightly associated to the intrinsically disordered N and C-terminal domains of the proteins^24^.

In metazoans, several conserved mechanisms allow cells to modulate their metabolism in response to a reduction in oxygen availability. Upon hypoxia, a first line of cellular responses involves a rapid reduction of ATP consumption. This relies on a strong inhibition of mRNA translation as protein synthesis is one of the most energy-consuming cellular processes. This initial adaptation phase to hypoxic conditions is usually described as “defensive” and is rapidly followed by a “rescue” phase where the gene expression program is largely remodeled in order to establish a prolonged tolerant state to hypoxia (reviewed in ref.^25^).

Knowing that TIS11/TTP proteins are short-lived factors, we hypothesized that the translational blockade observed in hypoxic cells would lead to a strong decrease in their cellular concentration and would in return influence the post-transcriptional regulation of gene expression upon variation of the oxygen in the cellular environment. Here, we tested this hypothesis by exploring the consequences of variations in oxygen concentration on dTIS11 protein levels and AMD in *Drosophila* S2 cells.

We observed that dTIS11 accumulation is highly sensitive to variations in oxygen concentration and contributes to gene expression reprogramming upon transition from a hypoxic to a reoxygenated environment. In particular, we demonstrated that TIS11 controls the level of lactate dehydrogenase (LDH) during reoxygenation and influences the metabolic adaptation of cells to oxygen variations.

Altogether, our data demonstrate that optimal metabolic adaptation to oxygen variations relies not only on regulation of gene transcription and enzyme activity but also on post-transcriptional mechanisms controlling mRNA stability such as AMD.

## Results

### Modulation of dTIS11 protein levels upon variations in oxygen concentration in *Drosophila* S2 cells

AMD is a major post-transcriptional mechanism regulating gene expression in eukaryotes and dTIS11 is an essential effector of AMD in *Drosophila*. Therefore, variations in dTIS11 levels are expected to result in major changes of the gene expression program. We have previously shown that proteins of the TIS11/TTP family are short-lived due to their rapid degradation by the proteasome^24^. Although phosphorylation protects these proteins from degradation, their intrinsic rapid turnover most probably contributes to the dynamics of AMD.

**Figure 1:**
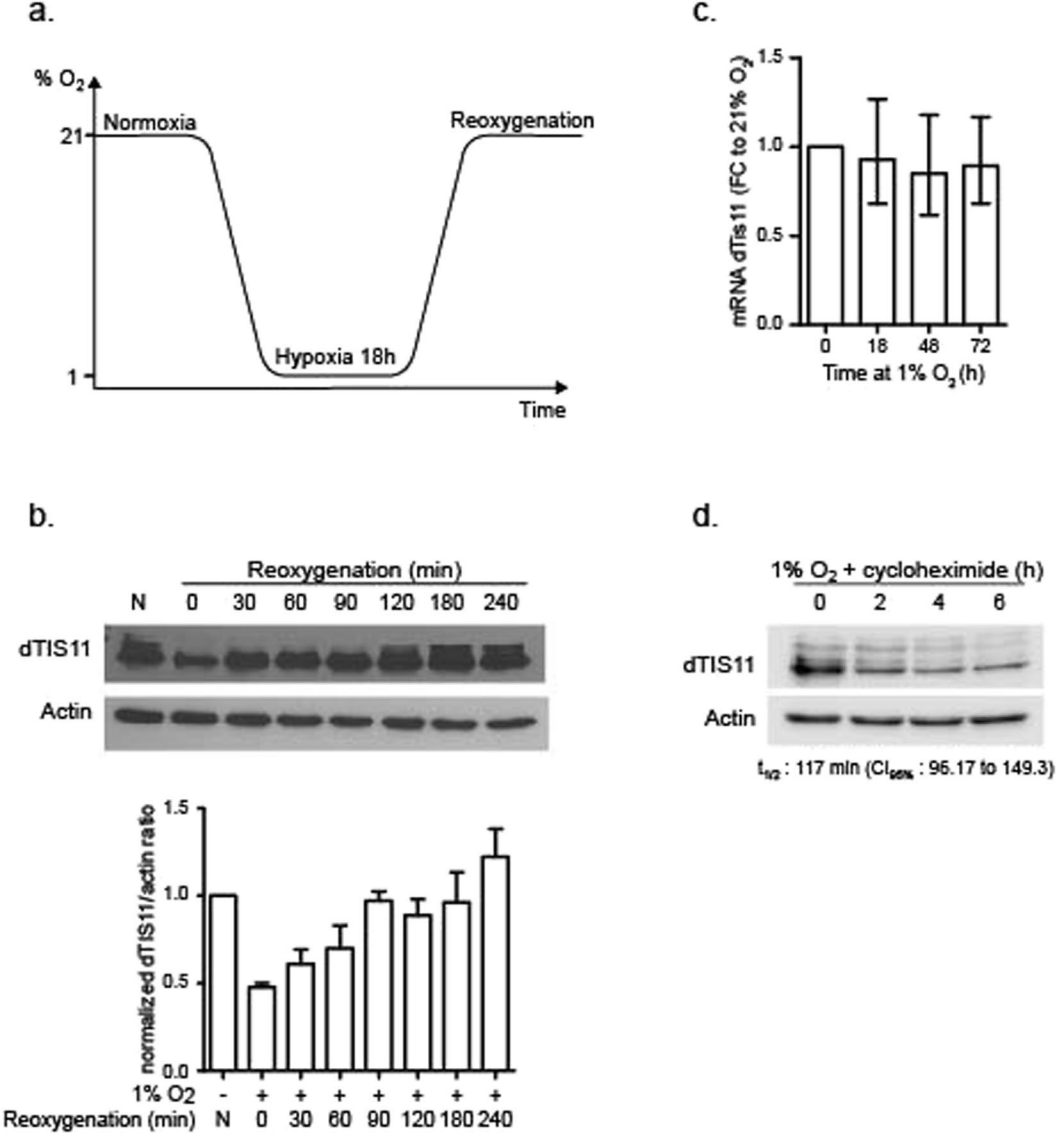
Influence of oxygen variations upon dTIS11 protein levels. (**a**) S2 cells were incubated at 21% (normoxia) or 1% O_2_ (hypoxia) for 18 hours then transferred back at 21% O_2_ for the indicated time. (**b**) Upper panel: Total protein extracts from S2 cells cultivated under indicated oxygen conditions were analyzed by Western Blot. Actin was used as endogenous control. Western Blot representative of >3 independent experiments. Lower panel: Quantification of 3 independent Western Blot. dTIS11/actin ratios were normalized on constitutive expression in normoxia. (**c**) Relative dTIS11 mRNA levels after increasing time in hypoxia. Total RNA was extracted and analyzed by qRT-PCR (n = 5) and normalized on the level of Rpl32. Geometric mean of fold change (FC) is shown. Error bar = C.I. 95%. Paired t-test on log2 FC. (**d**) dTIS11 and Actin were detected by western blot in cell extract from S2 cells incubated in 1% O_2_ for 18 h and treated with cycloheximide for the indicated time periods. dTIS11 half-life under these conditions was calculated based one the quantification of 3 independent experiments.

Oxygen is an essential component for aerobic organisms and variations in oxygen availability have a strong impact on cell metabolism and physiology. Severe oxygen reduction (hypoxia) induces a metabolic shift from cellular respiration towards anaerobic glycolysis and a marked reduction of energy consuming processes such as protein synthesis^25,26^. Accordingly, exposure of S2 cells to reduced oxygen concentration (1%) for 18 hours markedly down-regulates the association of mRNA to polysomes (Fig. S1), confirming a strong blockade of protein synthesis in S2 cells upon hypoxia^27^. We tested whether oxygen variations modified dTIS11 levels by incubating S2 cells for a prolonged time (18 h) at 1% oxygen followed by a reoxygenation phase at 21% oxygen (Fig. 1a). As shown in Fig. 1b, dTIS11 levels are strongly reduced by hypoxia but rapidly return to levels observed in normoxia upon reoxygenation while dtis11 mRNA accumulates at similar level in normoxic or hypoxic S2 cells (Fig. 1c). As previously described in normoxia^24^, we observed that dTIS11 protein has a short half-life in hypoxic S2 cells (t_1/2_ = 117 min.) (Fig. 1d). Therefore, the strong reduction of dTIS11 protein upon hypoxia is most likely due to rapid degradation combined with hypoxia-induced global arrest of protein synthesis.

Altogether, these results reveal that *dtis*11 expression is strongly and dynamically regulated by oxygen concentration, thereby suggesting that dTIS11-dependent AMD may contribute to gene expression reprogramming upon variations in oxygen levels.

### Transcriptome-wide analysis of hypoxia-induced genes containing ARE

Higher eukaryotes have developed coordinated mechanisms at both the transcriptional and post-transcriptional levels for optimal control of gene expression. Mechanisms governing the transcriptional shift upon oxygen deprivation have been well documented both in mammals and *Drosophila*^28,29^. By contrast, little is known on post-transcriptional controls contributing to cellular hypoxic response^30^. To evaluate the role of AMD in the control of gene expression in hypoxia, we performed a transcriptome-wide analysis of hypoxia-induced genes by RNA-sequencing (RNA-seq) of poly-A^+^ RNA of S2 control cells (see methods) incubated either at 21% or at 1% O_2_ for 18 hours.

**Figure 2:**
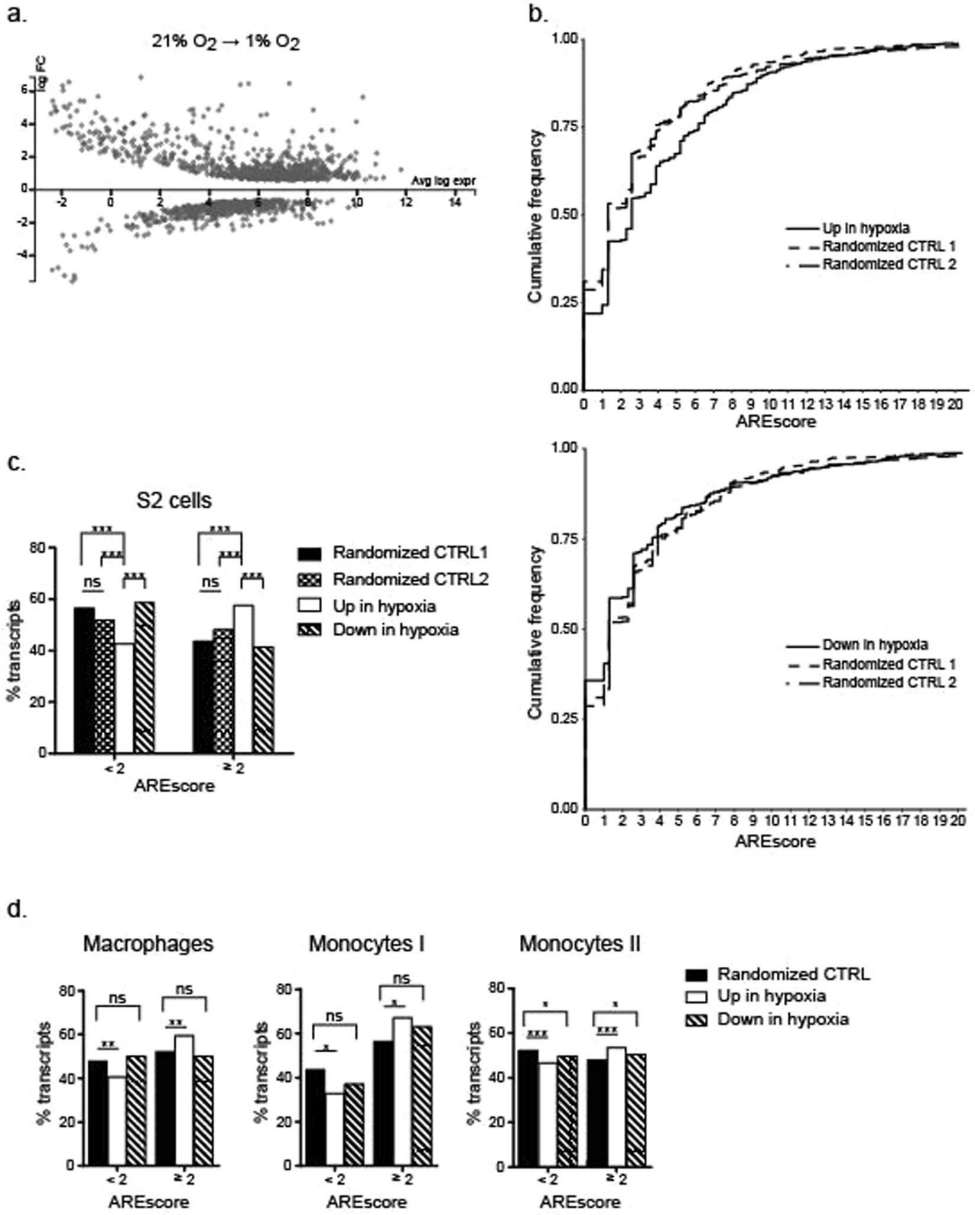
Hypoxia induced-transcripts are enriched in ARE. Total RNA from CTRL S2 cells in normoxia and hypoxia was analyzed by RNAseq. Differential gene expression analysis between hypoxia and normoxia was performed with the EdgeR method on the Degust website. FC > 1.5, FDR < 0.01 (**a**) MA plot showing the log_2_ FC and average log expression of the transcripts. (**b**,**c**) The AREScore of the upregulated or downregulated transcripts with FC > 1.5 (S2 hypoxia) was determined using the AREScore algorithm. Two randomly generated lists of respectively 2136 and 2175 transcripts expressed in S2 cells (Randomized CTRL 1 and 2) were used as control. (**b**) The cumulative frequency of transcripts of upregulated (upper panels) or downregulated (lower panels) was plotted according to their AREScore. Kolmogorov-Smirnov test was performed to determine the difference between the distributions of upregulated transcripts. (**c**) % transcripts with AREScore <2 or ≥2 in S2 cells in hypoxia compared to randomized CTRL.Results of Χ^2^ test (with Bonferoni correction) are: Up hypoxia Vs Down hypoxia p = 4.79 × 10^−16^; Up hypoxia Vs Random1 p = 4.85 × 10^−11^; Up hypoxia Vs Random 2 p = 9.04 × 10^−9^; Down hypoxia Vs Random1 p = 0.0305; Down hypoxia Vs Random2 p = 0.002; Random1 Vs Random p = 1). (**d**) AREScore analysis of genes up- and downregulated in hypoxia from publicly available data. Left panel: microarray analysis of RNA from human macrophages placed 24 h in hypoxia (NCBI Gene Expression Omnibus: GSE4630). Χ^2^ test: p-value = 0.009604 (upregulated transcripts); p-value = 0.5406 (downregulated transcripts). Middle panel: microarray analysis of RNA from human monocytes cultured 16 h in hypoxia (EMBL Array express archives: E-MEXP-445). Χ^2^ test: p-value = 0.02007 (upregulated transcripts); p-value = 0.2591 (downregulated transcripts). Right panel: RNAseq data from human monocytes enriched from PBMCs cultured for 48 h in hypoxia (European Nucleotide Archives PRJNA262464). Χ^2^ test: p-value = 1.591 × 10^−5^ (upregulated transcripts); p-value = 0.03494 (downregulated transcripts). A randomized control set of transcripts was generated from all the expressed genes for each experiment.

As previously shown for *Drosophila* adult and larvae^29,31^, hypoxia treatment also induces an acute change of the transcription profile in *Drosophila* S2 cells. Differential analysis reveals that expression of 695 and 456 genes is respectively up- or down-regulated more than 1.5-fold in hypoxia as compared to normoxia (Fig. 2a, Sup. Tables S1 and S2). To identify transcripts likely containing ARE, we calculated the AREScores^32^ of all potential isoforms reported in the NCBI RefSeq mRNA database for the up- and down-regulated gene subsets. These scores were compared to the AREScore values obtained from two lists of respectively 2136 and 2175 transcripts randomly generated from the list of genes expressed in S2 cells. We observed a significant difference in the distribution of AREScore frequencies between hypoxia-upregulated transcripts and both groups of randomly selected transcripts (Fig. 2b, upper panel) (Kolmogorov-Smirnov. CTRL1 p = 3.78 × 10^−10^, CTRL2 p = 6.66 × 10^−16^) suggesting that hypoxia-induced mRNA are more likely to contain ARE in their 3′UTR. Such difference was not observed when comparing the population of hypoxia downregulated transcripts with S2 expressed random transcripts (Fig. 2b, lower panel) (Kolmogorov-Smirnov. CTRL1 p = 0.252, CTRL2 p = 0.012).

The AREScore algorithm assigns a minimal score of 1 when detecting the presence of a single AUUUA pentamer in a 3′UTR^32^. The score increases with the number of pentamers, if pentamers are located within a region of high AU content or if pentamers are close to each other. These parameters reflect the fact that, both in invertebrates and mammals functional ARE frequently occur as multiple copies of close or overlapping AUUUA pentamers while a single copy of this motif is not sufficient to induce mRNA degradation (refs^7,8^, and references therein). An AREScore of 2 could therefore be considered as the minimal score required for a functional ARE. We compared the frequencies of transcripts with AREScores ≥2 (ARE-mRNA) and transcripts with scores <2 among transcripts induced in hypoxic conditions as compared to randomized controls (Fig. 2c). X^2^-test analysis revealed a significant statistical difference between these groups (see fig. legend).

In mammals, AMD regulates the stability of a wide range of transcripts and is essential to control the inflammatory response (ref.^33^ for review). mRNA decay mediated by dTIS11 homolog Tristetraprolin (TTP) plays a central role in modulating gene expression in activated myeloid cells^34–36^. Interestingly, hypoxia is also a major modulator of myeloid cells functions in physiological and pathophysiological environments^37,38^.

To evaluate the conservation of ARE enrichment in hypoxia-induced transcripts of myeloid cells, we performed the same analysis on publicly available microarray or RNAseq datasets from human primary macrophages^39^ and monocytes^40,41^. For each dataset, we established the AREScore of expressed genes as described above. We calculated the differential in gene expression for cells cultivated in normoxic or hypoxic conditions and defined for each experiment, groups of genes upregulated or downregulated more than 1.5-fold in hypoxia as compared to normoxia (see methods). Finally, we compared the frequencies of transcripts with AREScores ≥2 and transcripts with scores <2 among transcripts upregulated or downregulated in hypoxia to a randomized group of transcripts. For each experiment, X^2^-test analysis revealed a significant statistical difference between groups for upregulated transcripts while for groups of downregulated transcripts, little or no ARE enrichment was found when compared to randomized controls (Fig. 2d). Taken together, these data indicate that ARE are significantly enriched in mRNA expressed during hypoxia in human monocytes and macrophages, thereby suggesting an evolutionary conserved role of ARE in the control of hypoxia-induced genes from invertebrates to mammals.

### Hypoxic ARE-containing genes are downregulated upon reoxygenation in a dTIS11-dependent manner

**Figure 3:**
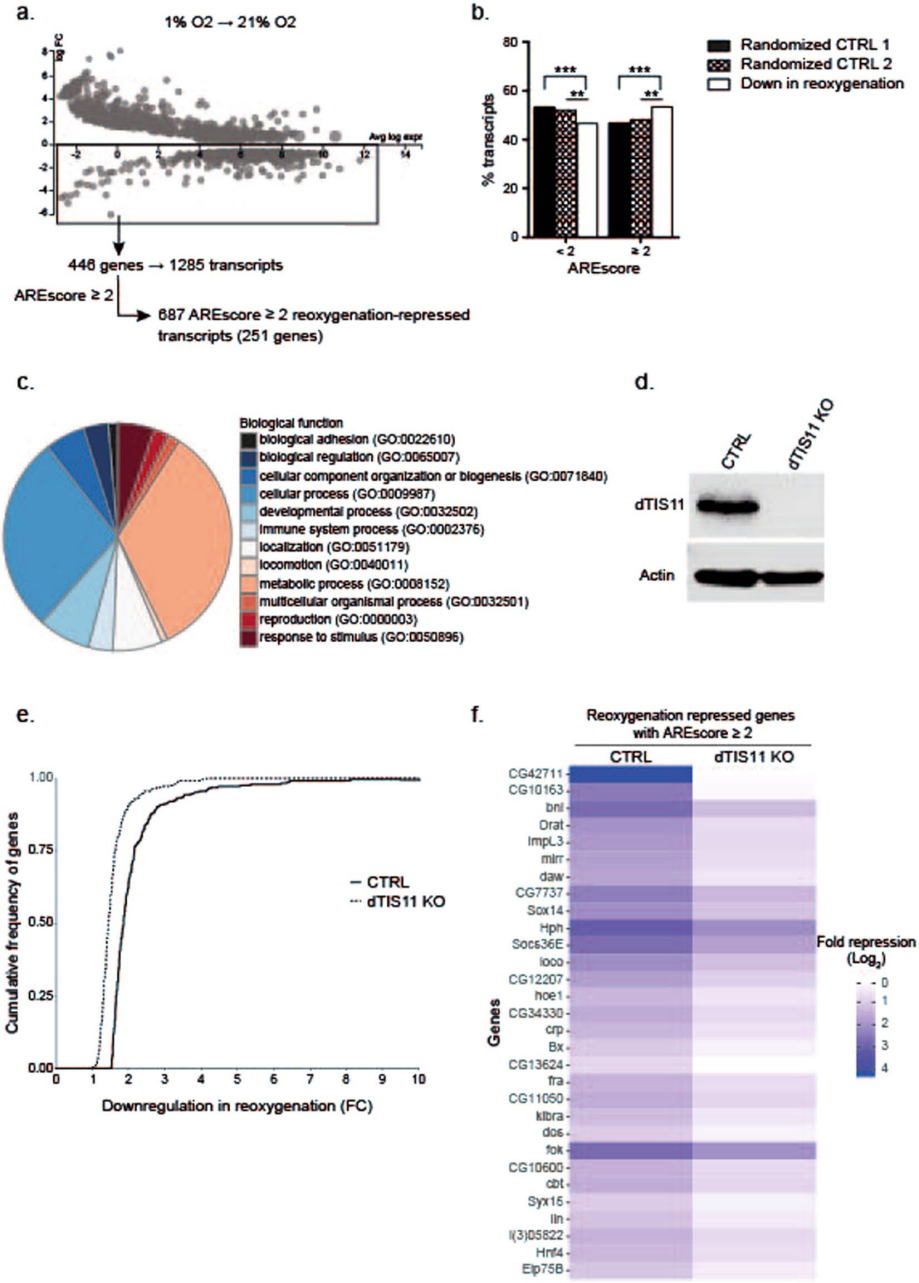
Hypoxic ARE-containing genes are downregulated upon reoxygenation in a dTIS11-dependent manner. (**a**) MA-plot of differentially expressed genes in CTRL cells upon reoxygenation with False Discovery Rate (FDR) < 0.05 and abs(FC) > 1.5. (**b**) % transcripts with AREScore <2 or ≥2 in S2 cells upon reoxygenation (90 min.) compared to 2 lists of randomized selected transcripts cells (Randomized CTRL 1 and 2). Results of Χ^2^ test are: Down in reoxygenation. Vs Random1 p = 5.94 × 10^−4^; Down in reoxygenation Vs Random2 p = 7.99 × 10^−3^. (**c**) GO term (Biological Processes) analysis of genes downregulated in CTRL cells upon reoxygenation (90 min.) after hypoxia. (**d**) Western Blot analysis of dTIS11 in CTRL and dTIS11 KO cells generated by CRISPR-Cas9 (see methods). (**e**) Total RNA extracted from dTIS11 KO and CTRL cells in hypoxia (18 h) and 90 min after reoxygenation were analyzed by RNAseq. Differentially regulated genes in hypoxia (1% O_2_, 18 h) and upon reoxygenation (21% O_2_, 90 min.) were analyzed by the EdgeR method for CTRL and dTIS11 KO cells. The cumulative frequency of transcripts detected as downregulated during reoxygenation was plotted according to their FC between hypoxia and reoxygenated conditions for both CTRL and dTIS11 KO cells. Kolmogorov-Smirnov test was performed to determine the difference between the distributions. (**f**) The AREScore of genes down-regulated in CTRL cells upon return to normoxia (FDR < 0.05) was determined using the AREScore algorithm. Heat map of log2 FC of the 30 most differentially expressed genes with AREScore ≥ 2 in CTRL and dTIS11 KO upon reoxygenation. See Sup. Table S3 for FC values of the complete gene list.

The rise in dTIS11 level upon transition from a hypoxic to a reoxygenated environment in S2 cells (see Fig. 1b) suggests that ARE-containing mRNA accumulated during hypoxia could be efficiently degraded upon reoxygenation. To investigate this hypothesis, we generated CRISPR-Cas9 clonal S2 cell lines inactivated for the *dtis11* or the *yellow* gene as control (CTRL)^42^. We first performed RNA sequencing on CTRL cells, cultivated in a hypoxic or reoxygenated environment. Detection of a negative fold change in gene expression in these conditions identifies genes with reduced transcription and/or increased mRNA degradation rate upon reoxygenation. This analysis identified 446 genes significantly downregulated upon reoxygenation (FDR < 0.05; FC < −1.5) (Fig. 3a). We selected from the RefSeq database the 1285 transcripts corresponding to these 446 genes and calculated their AREScores. We defined a group of 687 transcripts (from 251 genes) with AREScores ≥2 (Sup. Tables S3,S4). We compared the frequencies of transcripts with AREScores ≥ or <2 among transcripts repressed in reoxygenated conditions with two groups of randomly selected transcripts (Fig. 3b). X^2^-test analysis revealed a significant statistical difference between these groups (see fig. legend), suggesting that transcripts markedly repressed upon reoxygenation after hypoxia are more likely to contain ARE in their 3′ UTR. Gene Ontology (GO) term analysis showed that a majority of these ARE-containing mRNA repressed in reoxygenation are predicted to play a role in metabolic and cellular processes (Fig. 3c). Efficient downregulation of these mRNA in reoxygenation could therefore play a role in terminating the specific metabolic program established during hypoxia in metazoans^43^.

To evaluate the effect of dTIS11 on the reoxygenation-induced repression of ARE-containing mRNA, we performed a RNAseq experiment on hypoxic and reoxygenated dTIS11 KO cells (Fig. 3d) and calculated the differential of gene expression between these two experimental conditions (Sup. Tables S3, S4). We further compared the repression FC for ARE-containing mRNA (AREScore ≥2) upon reoxygenation in CTRL and dTIS11 KO cells. We calculated the cumulative frequencies in FC for this gene set both in WT and dTIS11 KO cells and observed a significant difference in these distributions (Fig. 3e) (Kolmogorov-Smirnov. P < 2.2 × 10^−16^). The heatmap shown in Fig. 3f displays the 30 (AREScore ≥2) genes showing the greatest difference in repression fold in CTRL and dTIS11KO cells. Taken together, these data indicate that the repression of ARE-containing mRNA upon reoxygenation is more efficient in CTRL cells than in dTIS11 KO cells, highlighting the repressive role of dTIS11 on ARE-containing mRNA during reoxygenation. Moreover, the association of this group of transcripts to metabolism suggests that dTIS11 is an important regulator for metabolic adaptation upon oxygen variations in *Drosophila*.

### ImpL3 mRNA stability is controlled by dTIS11-dependent AMD upon oxygen variations

Among ARE-containing mRNA induced by hypoxia (Sup. Table S1) and repressed upon reoxygenation in a dTIS11-dependent manner, we identified ImpL3 (Fig. 3f, Sup. Table S3). This gene encodes the *Drosophila* Lactate Dehydrogenase (LDH), an enzyme catalyzing the reduction of pyruvate into lactate coupled to the oxidation of NADH to NAD^+^. LDH plays a central role in the cellular adaptation to a hypoxic environment by contributing to the shift from oxidative to glycolytic metabolism (see ref.^28^ for recent review). However, the reduced yield of ATP production under glycolytic metabolism might have induced a selective pressure to favor an efficient and rapid downregulation of LDH gene expression upon return to normoxia after a hypoxic episode. We therefore investigated whether *ImpL3* could be actively down-regulated by dTIS11 during a hypoxic/normoxic transition in S2 cells.

**Figure 4:**
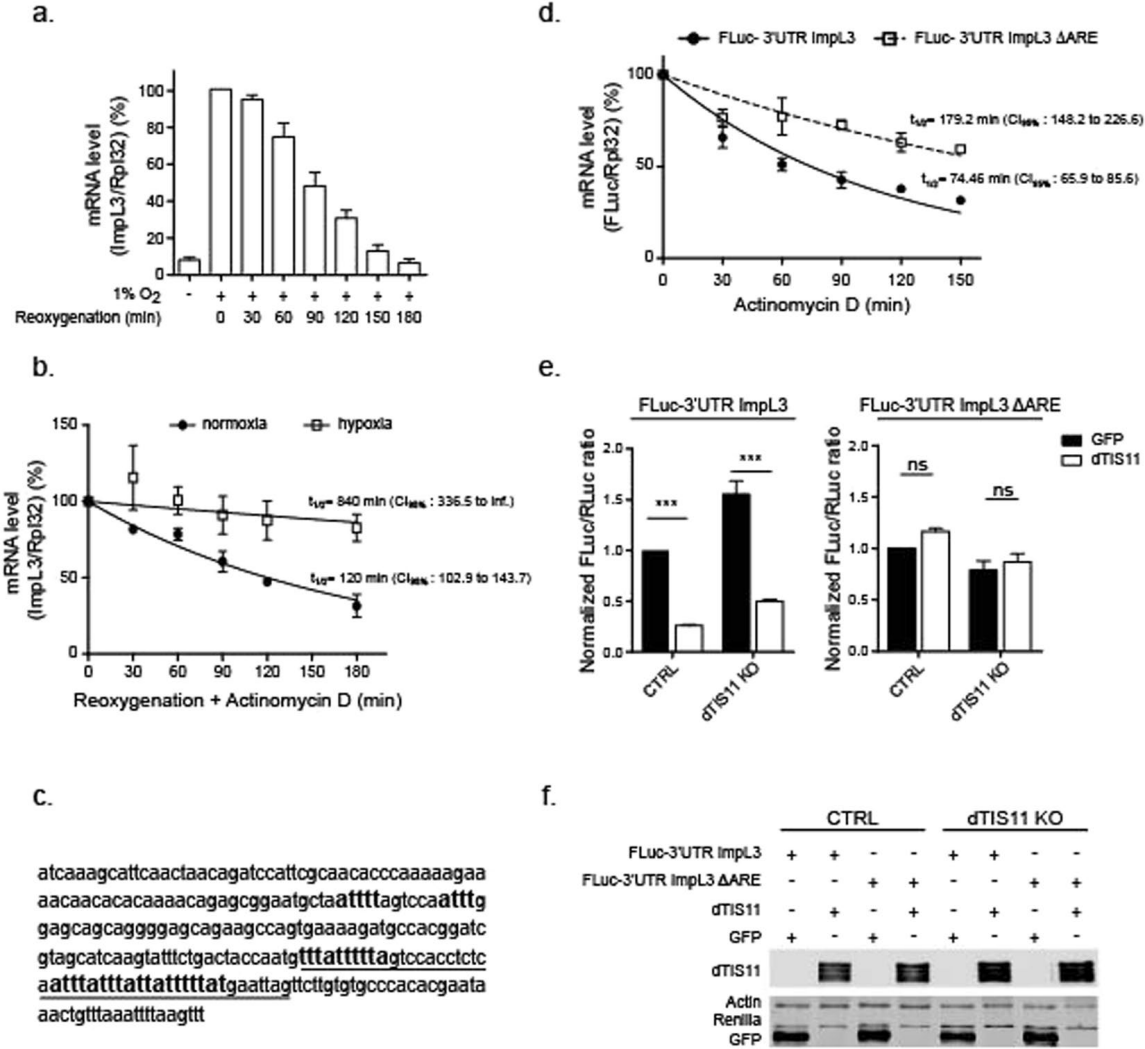
ImpL3 mRNA stability is regulated by oxygen variations through a TIS11 sensitive ARE (**a**) S2 cells were incubated at 1% O_2_ for up to 18 hours, then reoxygenated at 21% O_2_ up to 3 hours. ImpL3 mRNA was detected by Northern Blot (NB) and quantified by phosphorimager. ImpL3 mRNA level relative to Rpl32 was normalized on expression level measured at time = 0 after hypoxia for each biological replicate. Mean +/− SEM of 3 independent experiments. (**b**) ImpL3 mRNA stability in normoxia and hypoxia. S2 cells were placed at 1% O_2_ for 18 hours, then treated with actinomycin D (5 µg/ml) and placed at either 21% or 1% O_2_ for up to 180 min. Cells kept at 21% O_2_ were used as negative control for ImpL3 induction (Ctrl). Mean +/− SEM of 3 (hypoxia) or 2 (normoxia) independent experiments. (**c**) 3′UTR of ImpL3 mRNA. ARE are underligned. (**d**) Expression of FLuc- 3′UTR ImpL3 or FLuc-3′UTR ImpL3 ∆ARE reporter genes was induced in stably transfected S2 cells by CuSO_4_ (0.5 mM) for 3 hours. Cells were then treated with actinomycin D (5 µg/ml) for the indicated times. Total RNA was extracted and FLuc mRNA was detected by NB and quantified by phosphorimager. To determine reporter mRNA half-lives, FLuc mRNA level relative to Rpl32 was normalized to expression levels measured before actinomycin D addition. Mean ± SEM of 3 independent experiments. (**e**) FLuc reporter genes containing full-length or ARE-deleted ImpL3 3′UTR were transiently co-transfected with a control RLuc-V5 plasmid and either GFP-V5 or dTIS11-V5 expressing plasmids in CTRL and dTIS11 KO cells. V5-tagged Renilla, GFP and dTIS11 were detected in western blot with an anti-V5 antibody, actin was detected as in Fig. 3d. (**f**) Dual luciferase assay was performed on the corresponding cell lysates after 18 h CuSO_4_ (0.5 mM) treatment to induce expression. FLuc/RLuc ratios were determined for each condition. Mean ± SEM of 3 independent experiments. 2-way ANOVA and Bonferroni’s post-test: ns: p > 0.5, ***p < 0.001.

*ImpL3* expression was analyzed upon oxygen variation in S2 cells. The production of lactate dehydrogenase is detectable after 24 h and reaches a steady state level after 48 hours in 1% O_2_ (not shown). We then measured the expression of *ImpL3* upon re-oxygenation after 18 hours of hypoxia and observed that ImpL3 mRNA decreased to basal levels after a short time period (~180 min) (Fig. 4a). Analysis of ImpL3 mRNA half-life in hypoxia and upon reoxygenation revealed that ImpL3 mRNA is strongly destabilized upon return to normoxia as compared to hypoxia (Fig. 4b). These results indicate that *ImpL3* expression is highly induced upon oxygen deprivation but is rapidly shut off by mRNA destabilization upon increasing oxygen concentrations.

Analysis of *ImpL3* sequence revealed the presence in its 3′UTR of several AUUUA pentamers in U-rich context (Fig. 4c). To test the functional role of these motifs as mRNA destabilizing elements, we generated two reporter genes in which the Firefly luciferase (FLuc) coding sequence was placed under the control of the metallothionein promoter and flanked by ImpL3 3′UTR containing or not ARE (see methods). These constructs were transfected in S2 cells and transcription of the reporter genes was induced by incubation of the cells with copper sulfate. The half-life of FLuc mRNA reporters was determined by northern blot, upon transcription inhibition by Actinomycin D. We observed that FLuc mRNA bearing wild-type ImpL3 3′UTR had a markedly shorter half-life as compared to FLuc containing ImpL3 3′UTR devoid of ARE (Fig. 4d). To test the implication of dTIS11 in ARE-dependent ImpL3 mRNA destabilization, the above mentioned FLuc reporter genes were co-transfected with plasmids expressing either GFP or dTIS11 and a Renilla luciferase control vector (RLuc) in CTRL and dTIS11 KO S2 cells (Fig. 4f). As shown in Fig. 4e, the FLuc/RLuc ratio is markedly reduced for the FLuc gene with the complete ImpL3 3′UTR (left panel) upon overexpression of dTIS11 as compared to GFP control and FLuc/RLuc ratio is increased in dTIS11 KO cells as compared to FLuc/Rluc ratio in CTRL cells. Moreover, overexpression of dTIS11 in dTIS11 KO cells rescues the mutant phenotype by restoring a marked downregulation of the FLuc reporter mRNA containing ImpL3 ARE. These effects are specific to the presence of the ARE in the reporter gene as FLuc/RLuc ratio for the FLuc reporter lacking ImpL3 ARE is similar upon overexpression of dTIS11 and GFP both in CTRL or dTIS11 KO cells (Fig. 4e, right panel). Altogether, these results demonstrate that the AU-rich motifs present in *ImpL3* 3′UTR are bona fide ARE promoting mRNA decay in a dTIS11-dependent manner in normal oxygen concentrations.

### dTIS11 binds ImpL3 ARE and destabilizes ImpL3 mRNA upon return to normoxia

**Figure 5:**
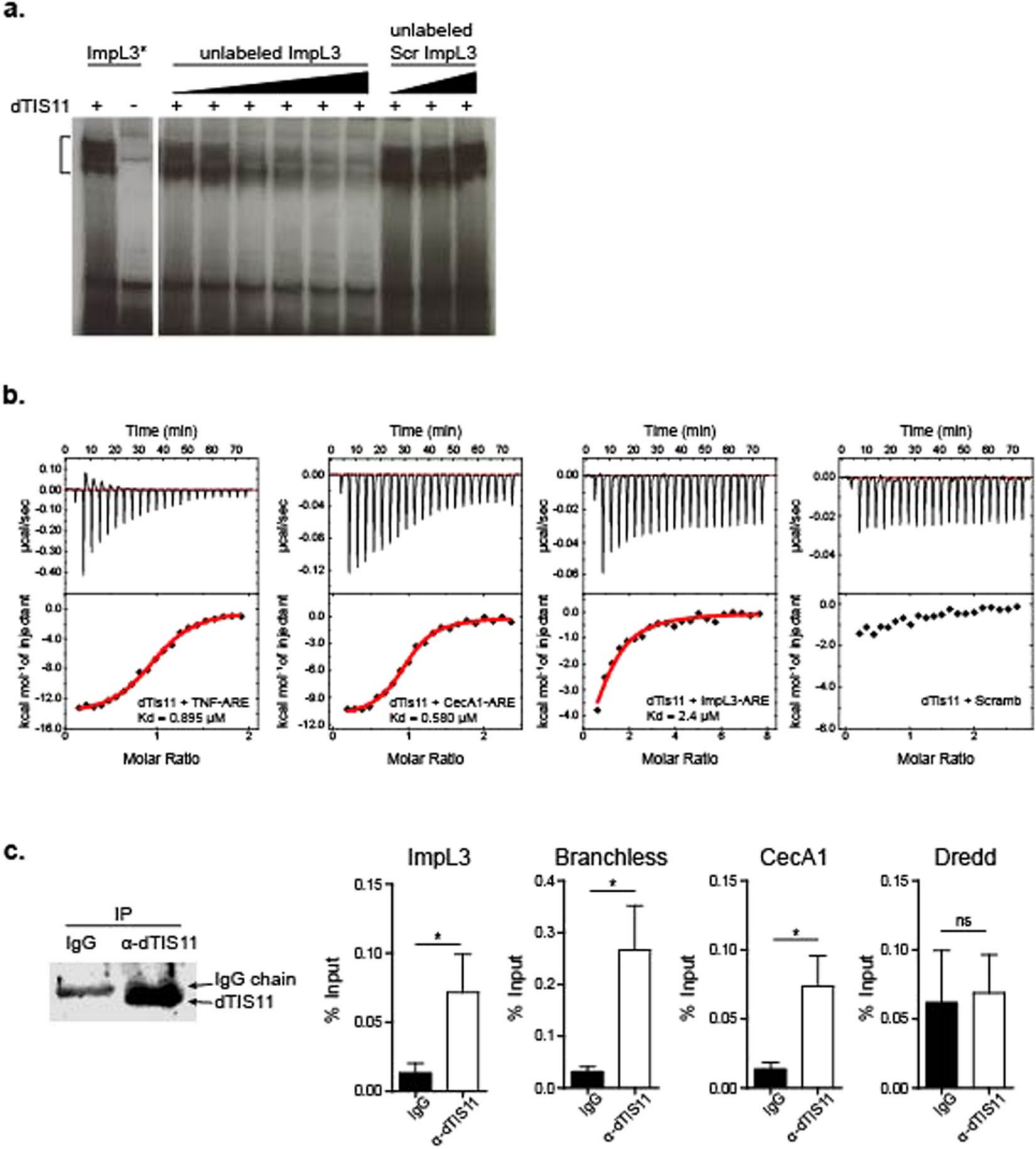
dTIS11 binds ImpL3 ARE. (**a**) ^32^P-labeled RNA probes of ImpL3 ARE (ImpL3) or scrambled ImpL3 sequence (Scr ImpL3) were transcribed *in vitro* and incubated with recombinant dTIS11 protein for EMSA. dTIS11-binding specificity was assessed by competition with increasing amounts of unlabeled ImpL3 ARE or scrambled ImpL3 RNA: 4-, −6, −10, −12, −20, 25-fold molar excess of ImpL3 ARE and 6-,10-, 20-fold molar excess of scrambled ImpL3. EMSA representative of 3 independent experiments. ImpL3*: ^32^P-labeled ImpL3 probe. (**b**) Binding of dTIS11 to different RNA fragments. From left to right: ITC titration of dTIS11 into TNF-ARE, CecA1-ARE, ImpL3-ARE and an RNA fragment with a scrambled sequence of the same length as ImpL3-ARE probe. All the titrations were done at 10 °C. (**c**) S2 cells were incubated in hypoxia (1% O_2_) for 18 h and returned to normoxia (21% O_2_) for 60 min. before lysis. IP was carried out with anti-dTIS11 monoclonal antibody or murine IgG1 as negative control. Precipitation of dTIS11 was evaluated by Western blot analysis (upper panel, full size image presented in Supplementary Figure S2). Total RNA was extracted from the immunoprecipitation pellet by the Trizol method and the levels of indicated transcripts were measured in triplicates by RT-qPCR. Results are shown as % Input. Mean ± SEM of 5 independent experiments.

To test the capacity of dTIS11 to directly bind ImpL3 ARE, we performed electrophoretic mobility shift assays (EMSA) with a labeled RNA probe corresponding to ImpL3 ARE-enriched region (Fig. 5a). Incubation of ImpL3 probe with recombinant dTIS11 protein resulted in a band shift of the ImpL3 probe. This band shift was efficiently competed by increasing amounts of ImpL3 unlabeled RNA and but not by similar amounts of unlabeled RNA corresponding to a scrambled ImpL3 sequence (see methods). We further compared the binding of dTIS11 to different RNA probes by Isothermal Titration Calorimetry (ITC)^44^. As shown in Fig. 5b, the complex formed between dTIS11 and an RNA sequence corresponding to Impl3 ARE has a measured dissociation constant of Kd = 2.4 µM. In the same experimental conditions, dTIS11 binds to previously described targets TNF ARE^45^ and CecA1-ARE^22^ with Kd of 0.895 and 0.580 µM, respectively. No binding was detected to a scrambled sequence of the ImpL3-ARE.

To confirm the binding of dTIS11 to ImpL3 mRNA *in vivo*, S2 cells were cultivated in hypoxia for 24 h before reoxygenation for 60 min. Cell extract was immunoprecipitated using anti-dTIS11 antibody^46^ or control IgG (Fig. 5c, left panel). Pelleted RNA was purified, and mRNA quantification was performed by RT-qPCR. Similarly to CecA1 and Branchless, two *bona fide* dTIS11 mRNA targets, Impl3 mRNA was reproducibly enriched by immunoprecipitation with anti-dTIS11 antibody relatively to control IgG. The Dredd mRNA, which is devoid of ARE and expressed at similar levels in hypoxic and normoxic conditions (not shown), was not or only minimally enriched by dTIS11 immunoprecipitation in these conditions (Fig. 5c, right panel). Altogether, these results indicate that dTIS11 directly binds to ARE present in ImpL3 mRNA 3′UTR.

**Figure 6:**
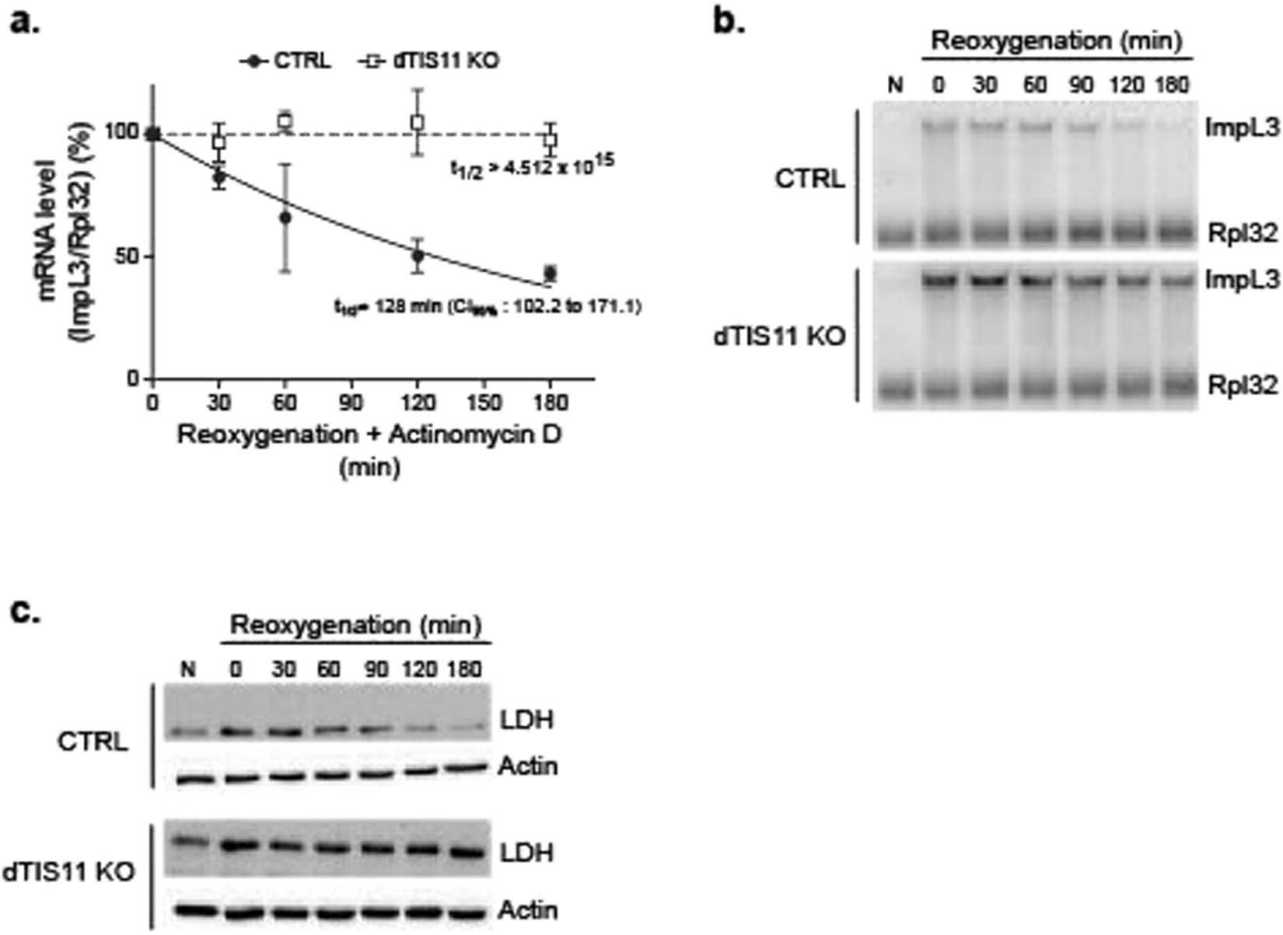
dTIS11 destabilizes ImpL3 mRNA upon return to normoxia. (**a**,**b**) dTIS11 KO and CTRL cells were placed 18 h at 1% O_2_ before reoxygenation at 21% O_2_ for the indicated times. (**a**) dTIS11 KO and CTRL cells were treated with actinomycin D (5 µg/ml) for the indicated times prior to RNA extraction. For the half-life of ImpL3 mRNA, NB were quantified by phosphorimager and ImpL3 expression relative to Rpl32 was normalized to expression levels at time = 0 at 21% O_2_ after hypoxia. Mean +/− SEM of 3 independent experiments. (**b**) Total RNA was extracted and ImpL3 mRNA was detected by Northern blot. Rpl32 was used as endogenous control. NB representative of 3 independent experiments. (**c**) WB analysis of LDH and dTIS11 in dTIS11 KO and CTRL cells. Total protein extraction was performed at the indicated time points upon return to normoxia. Actin was measured as endogenous control. Different acquisition times were used for LDH and Actin antibody.

RNA sequencing data revealed that ImpL3 mRNA accumulation is increased upon *dtis11* inactivation in S2 cells upon oxygen recovery (Fig. 3e). We analyzed ImpL3 mRNA accumulation in CTRL and dTIS11-deficient S2 cells upon reoxygenation after 18 h in hypoxia. As shown in Fig. 6b, depletion of dTIS11 markedly increases ImpL3 mRNA levels upon return of the cells to high oxygen concentrations. This increase of ImpL3 mRNA is due to its stabilization upon dTIS11 depletion as ImpL3 mRNA half-life is markedly increased upon *dtis11* inactivation (Fig. 6a). Consequently, the increased accumulation of ImpL3 mRNA in dTIS11 KO cells leads to a prolonged accumulation of lactate dehydrogenase after return to normoxia (Fig. 6c). Altogether, these results demonstrate a direct role of dTIS11 in the down-regulation of *ImpL3* gene expression upon return of cells to normoxia.

### dTIS11 controls energy metabolism and cell proliferation upon reoxygenation after hypoxia

**Figure 7:**
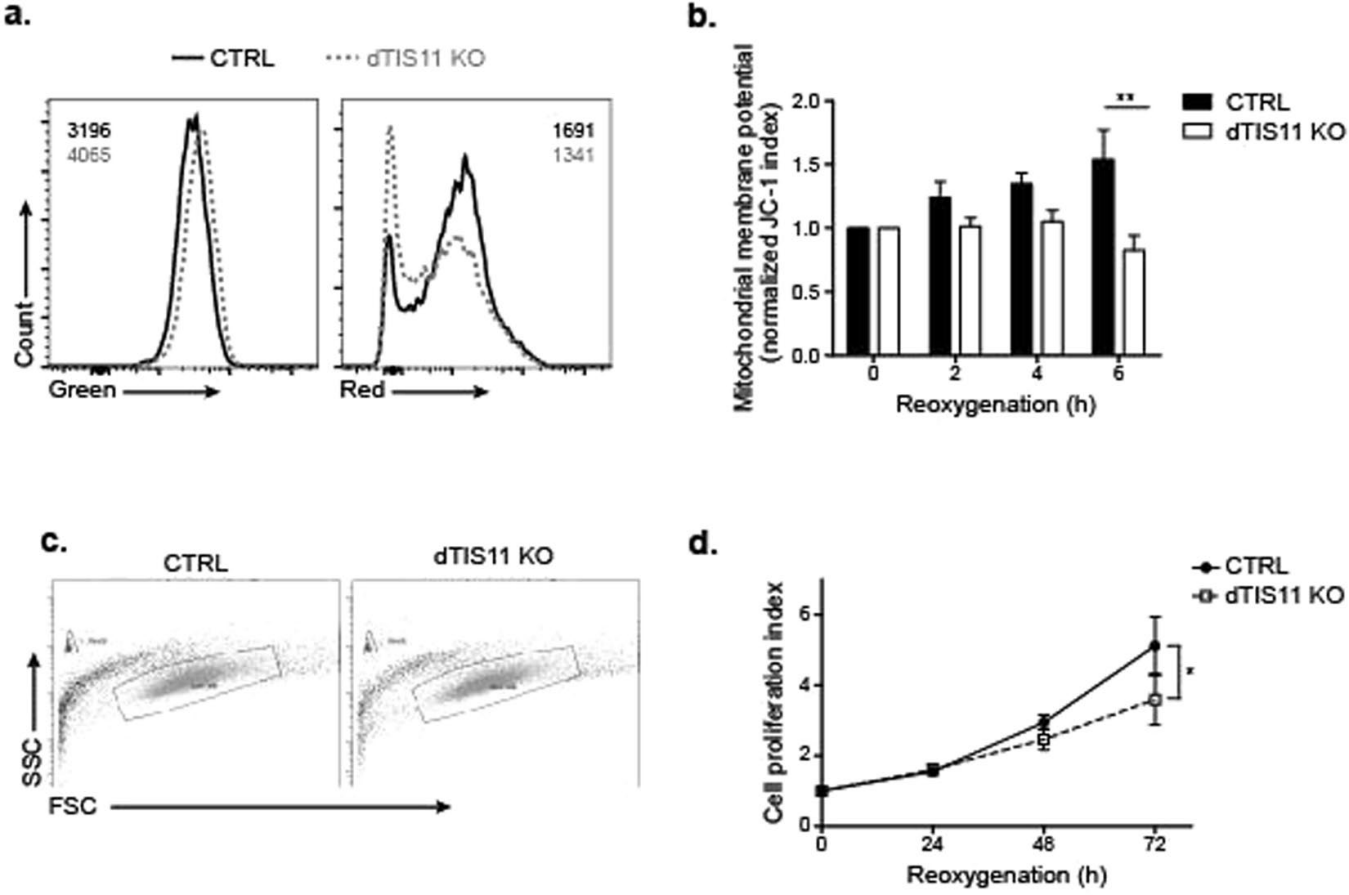
dTIS11 deficiency leads to impaired mitochondrial polarization and proliferation upon return to normoxia. dTIS11 KO and CTRL cells were placed at 1% O_2_ for 48 h then reoxygenated at 21% O_2_ for the indicated times. (**a**,**b**) Cells were collected at the indicated time points and incubated with JC-1 (2 µM) prior to analysis by flow cytometry. Mitochondrial depolarization is indicated by a decrease in the red/green ratio. (**a**) Representative FACS plots for green and red fluorescence for dTIS11 KO (grey, dotted line) and CTRL (black, full line) at 6 hours after return to normoxia. Mean fluorescence intensities (MFI) are indicated on the graph. Results are representative of 3 independent experiments. (**b**) JC-1 index was normalized to JC-1 index at 0 h return to normoxia for both cell lines. Mean +/− SEM of 3 independent experiments. 2-way ANOVA and Bonferroni’s post-test showed an overall difference between cell lines with p < 0.01 and a significant difference at 6 hours in particular. **: p < 0.01. (**c**,**d**) Cells were collected at the indicated time points and counted by flow cytometry using Polybeads. (**c**) Representative gating strategy showing the beads (upper left corner gate) and live cells (center gate), showing the similar size and granulosity of dTIS11 KO (lower plot) and CTRL (upper plot) cells before hypoxia. FACS was stopped after 500 beads. (**d**) Cells were counted at the indicated time points and normalized to the number of cells after 48 h hypoxia to yield the proliferation index for each cell line. Mean +/− SEM of 4 independent experiments. 2-way ANOVA and Bonferroni’s post-test showed an overall difference between cell lines with p < 0.05.

Prolonged accumulation of lactate dehydrogenase in dTIS11-deficient cells upon oxygen recovery led us to investigate whether dTIS11 inactivation would hamper resumption of oxidative phosphorylation and cell proliferation upon oxygen supply. To monitor oxidative phosphorylation, we evaluated the mitochondrial membrane potential by performing a kinetic analysis of JC-1 staining of CTRL and dTIS11 KO upon oxygen recovery (ref.^47^ and methods). As shown in Fig. 7a,b, JC-1 staining index steadily increased upon oxygen recovery in CTRL cells while it remained at basal levels in dTIS11 KO cells, thereby indicating that dTIS11 expression is necessary for the onset of oxidative phosphorylation upon oxygen supply.

**Figure 8:**
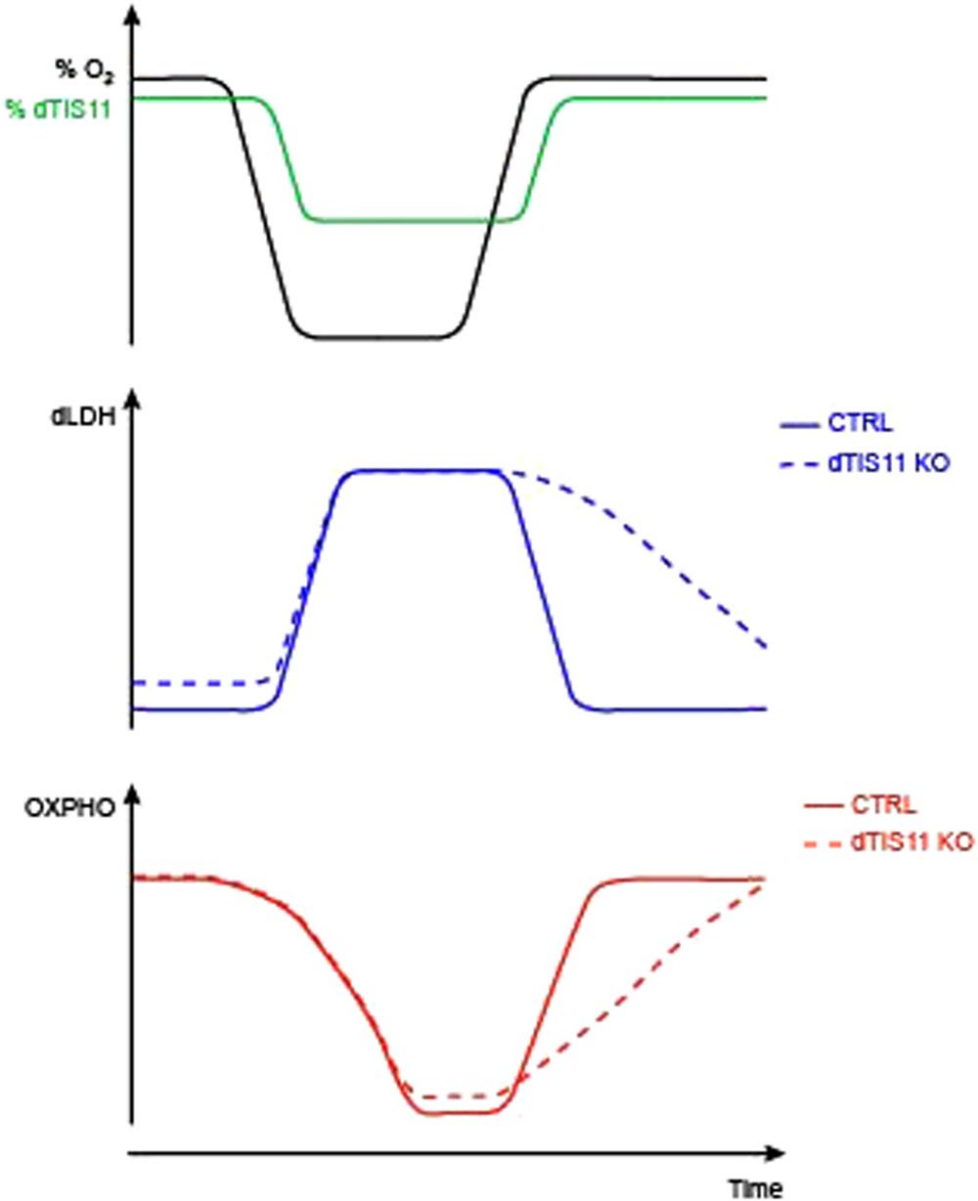
Model recapitulating dTIS11-dependent AMD post-transcriptional control upon oxygen variations.

We also compared the proliferation capacity of CTRL and dTIS11-deficient S2 cells upon oxygen recovery by cell counting using beads-standardized flow cytometry (see methods). As shown in Fig. 7c,d, CTRL cells resumed proliferation more efficiently than dTIS11 KO cells, further revealing the importance of dTIS11 for optimal proliferative capacity of *Drosophila* cells upon return to normoxic conditions. In summary our data indicate that fluctuations in dTIS11 level observed in Drosophila S2 cells exposed to variations in oxygen concentrations contribute to the stabilization of ARE-containing mRNA during hypoxia and destabilization upon reoxygenation. This mechanism regulates the level of LDH during the transition from a hypoxic to a reoxygenated environment and favors the return to an oxidative phosphorylation-based metabolism (Fig. 8).

## Discussion

AMD is a major evolutionary conserved mechanism controlling gene expression at the post-transcriptional level. We and others have shown that the unique member of the TIS11 protein family acts as a master *trans*-acting factor of AMD in *Drosophila*^21,22,32,45^. In this study, we demonstrate that dTIS11 protein level greatly fluctuates upon oxygen variations in *Drosophila* S2 cells. Indeed, dTIS11 protein accumulation is strongly decreased upon oxygen deprivation, most probably as a consequence of general translational blockade combined to dTIS11 rapid turnover by the 20 S proteasome^24^. Reversibly, dTIS11 rapidly returns to basal levels upon return to ambient oxygen concentration.

Oxygen is central to the energy production of aerobic organisms and variation of oxygen supply induces strong modifications in the cell metabolic program^43^. The response of hypoxia tolerant systems to oxygen deprivation occurs in two phases that can be considered as defense and rescue processes. The first lines of defense against hypoxia include a balanced suppression of ATP demand and ATP supply pathways; this regulation stabilizes adenylate concentrations at new steady-state levels as ATP turnover rates greatly decline. Energy-consuming processes such as ion pumping and protein synthesis are down-regulated^26^. The latter process results from a rapid and massive polysome disassembly leading to translational arrest (ref.^48^ and Fig. S1). The secondary rescue mechanisms include adaptation of the metabolic program and rely on major changes in the gene expression program mainly under control of the HIF family of transcription factors^28,43,49^.

Here, we show that dTIS11 level is markedly decreased upon hypoxia and is followed by a rapid increase upon return to normoxia (Fig. 1b), suggesting that control of mRNA stability contributes to the molecular dynamics adapting gene expression to oxygen availability. This hypothesis is strengthened by a significant enrichment of ARE-containing mRNA in hypoxia-upregulated transcripts in *Drosophila* S2 cells as measured by the AREScore algorithm (Fig. 2b,c). We evaluated the efficiency of mRNA clearance during reoxygenation by comparing global mRNA levels in hypoxic and reoxygenated S2 cells (Fig. 3a). This analysis revealed an ARE enrichment for mRNA downregulated during reoxygenation (Fig. 3b). Moreover, efficient clearance of ARE-containing mRNA upon reoxygenation requires dTIS11 as demonstrated by differences in FC upon reoxygenation between dTIS11 KO and CTRL cells (Fig. 3e). Taken together, these observations support that dTIS11 could enhance the clearance of ARE-containing mRNA upon cells reoxygenation after a hypoxic episode. This mechanism could favor the disappearance from the cytoplasm of mRNA synthesized during hypoxia but which could be detrimental to the cell if maintained at a high level when oxygen concentration returns to a normoxic level. Gene ontology analysis performed on ARE-containing mRNA repressed upon reoxygenation shows a preponderance of transcripts involved in cellular metabolism (Fig. 3c), suggesting an important role of AMD in the tight and dynamic control of metabolism for cellular adaptation to oxygen variations. This hypothesis is further sustained by the identification of ImpL3, Cabut (cbt) and Hnf4 transcripts, all shown to control metabolism and glucose homeostasis^50–52^ in the list of 30 ARE-containing mRNA whose repression upon reoxygenation is most sensitive to dTIS11. This subgroup of transcripts also includes branchless (bnl) and cropped (crp) which play major roles in the morphogenesis of the tracheal (respiratory) system (Fig. 3e)^53,54^. Of note, *bnl* is transcriptionally controlled by HIF^55^ and was previously identified as a target of dTIS11^21^.

The analysis of publicly available transcriptomic data from human monocytes/macrophages under normoxia or hypoxia revealed an increase in ARE frequency in hypoxia-induced mRNA (Fig. 2d), suggesting that ARE enrichment in hypoxia-induced mRNA could be evolutionary conserved. The influence of post-transcriptional controls of gene expression under hypoxic conditions in mammals has been explored previously (reviewed in ref.^30^). In contrast, the contribution of post-transcriptional control of gene expression during reoxygenation after a hypoxic episode remains poorly explored. Hence, several observations suggest that the dynamic control of mRNA stability contributes to the return to a normoxic gene expression program after hypoxia. First, we previously described that TTP, a mammalian member of the TIS11 protein family dTIS11 is degraded by a similar mechanism as dTIS11^24^, suggesting that the level of TIS11/TTPs could also fluctuate in response to variations in oxygen concentration in mammalian cells similarly to dTIS11 in *Drosophila* S2 cells. In accordance with this hypothesis, TTP has been shown to downregulate several hypoxia-induced mRNA in mammalian cells and could therefore contribute to the termination of a hypoxic response^56–58^. Genes encoding mammalian TTP/TIS11 proteins are controlled by complex cell type-specific regulatory networks^10,36,59^. Therefore, the regulatory activity of these proteins during a hypoxic or reoxygenation episode could be influenced by several other parameters than their *Drosophila* dTIS11 counterpart. Moreover, the emergence of a larger ARE-BP repertoire in mammals most probably contributed to the complexification of AMD under hypoxic conditions^5,7^. It is worth noting that HuR, an ARE-BP with mRNA stabilizing effect, was observed to migrate from the nucleus to the cytoplasm in hypoxia and could therefore contribute to the stabilization of ARE-containing mRNA during hypoxia^60,61^.

The importance of AMD as a mechanism controlling gene expression during the transition from a hypoxic to reoxygenated environment is illustrated by our observation that *ImpL3*, which encodes the unique Lactate Dehydrogenase in *Drosophila*, contains ARE (AREScore = 4.1) and is highly regulated upon oxygen variations. We show that ImpL3 mRNA is rapidly degraded upon reoxygenation (Fig. 4a,b) in a dTIS11-dependent manner (Fig. 6a,b). Moreover, reporter and binding assays show that ImpL3 AU-rich elements act as *bona fide* ARE recruiting dTIS11 to promote mRNA degradation (Figs 4d,e and 5). Our results also reveal that dTIS11 deficiency leads to prolonged accumulation of ImpL3 mRNA and LDH upon reoxygenation (Fig. 6c) and impairs resumption of oxidative phosphorylation and cell proliferation upon oxygen supply (Fig. 7).

Expression of lactate dehydrogenase (LDH) is central for adaptation of the cellular metabolic program to an oxygen-depleted environment. The tetrameric LDH enzymes catalyze the conversion of pyruvate to lactate and regenerate NAD + co-factor previously reduced to NADH during the glycolytic ATP producing phase^62^. During *Drosophila* embryonic development, *ImpL3* expression is upregulated upon oestrogen-related receptor signaling during mid-embryogenesis to sustain larval growth^63^. Moreover, *in situ* hybridization shows that ImpL3 transcripts are detected as early as stage 11 and are abundantly expressed in developing muscles from stage 13 to become predominantly muscular at stage 15. Loss of function of *ImpL3* strongly impairs muscle development, suggesting that the conversion of pyruvate into lactate is an important metabolic pathway for this developmental program^64^. One can speculate that during development, increased levels of LDH contribute to aerobic glycolysis (Warburg effect) used by highly proliferating cells to sustain synthesis of macromolecules. It would be interesting to analyze the role of dTIS11 in the control of *ImpL3* expression during development as *dtis11* KO flies although viable, display a 24 h eclosion delay^45^.

Most importantly, in *Drosophila*, all tissues are exposed to ambient O_2_ pressures^65^ and cells have thus to adapt rapidly to ambient oxygen variations. Therefore, we might speculate that AMD-mediated regulation of *ImpL3* expression contributes to the rapid return to oxidative phosphorylation in normoxia to ensure the return to maximal ATP yield to sustain the organism homeostasis^66^. Noteworthy, ARE are conserved in *ImpL3* gene within *Drosophila* genus (data not shown) whose cells are all exposed to ambient oxygen pressures and have to rapidly adapt to oxygen variations.

In mammals, oxygen deprivation leads to the activation of *ldha* gene which encodes the A subunit of lactate dehydrogenase. *Ldha* gene is also up-regulated upon aerobic glycolysis and is a hallmark of cell proliferation and cancer progression (Warburg effect) (ref.^67^ for review). Transcription of *Drosophila ImpL3* and mammalian *ldha* appears to be regulated by highly conserved mechanisms. Indeed, both are induced by HIF1 upon oxygen deprivation^68^ and are upregulated by c-myc^69,70^. Several reports indicate that rat LdhA mRNA is unstable due to the presence of destabilizing elements in its 3′UTR and that Ldha mRNA is stabilized upon protein kinase A activation by the binding of a protein complex to an AU-rich cis-acting element in Ldha 3′UTR^71,72^.Therefore, it appears that post-transcriptional regulation of lactate dehydrogenase has been conserved across evolution, although the *cis-* and *trans*-acting elements might have diverged from insects to mammals with the diversification of the post-transcriptional determinants across evolution.

In conclusion, our study identifies ARE-mediated decay as a new regulatory mechanism contributing to gene expression reprogramming during normoxia/hypoxia transitions and suggests that global inhibition of protein synthesis influences the expression of specific gene subsets containing ARE by modulating the level of the post-transcriptional regulator dTIS11.

## Material and Methods

### Reagents

Actinomycin D and DNA oligonucleotides were purchased from Sigma-Aldrich. Puromycin, hygromycin were purchased from InvivoGen. pAC-sgRNA-cas9 and pAC-y1sgRNA-cas9 plasmids were obtained from Addgene (# 49330 and 49331). Anti-LDH (H-160) antibody was purchased from Santa Cruz (sc-33781). Anti-actin (A2066) antibody was purchased from Sigma-Aldrich. dTIS11 monoclonal antibody was produced as previously described^46^.

### Cell culture and transfection

Non-adherent *Drosophila* S2 cells were kindly provided by Neal Silverman (Boston, USA). Cells were maintained in Schneider’s *Drosophila* medium (Genaxxon bioscience) supplemented with 10% HyClone FBS (Perbio) at 24 °C. Hypoxia (1% O_2_) was achieved by displacing oxygen in a hypoxia chamber with purified N_2_ gas whose flow was controlled by an oxygen sensor (CoyLab).

Transfections were performed with Fugene HD according to the manufacturer’s instructions (Roche). Stable cell lines transfected with the dTIS11-targeting pspCas9-puro were generated by selecting transfected cells with puromycin (5 µg/ml) for at least 3 weeks.

Cells were further subcloned by limit dilution in 100 µl medium supplemented with S2 cell-conditioned medium (20%) and puromycin. Stable cell lines transfected with pMT constructs in combination with a hygromycin-resistant plasmid (1/20 ratio) were selected with hygromycin (200 µg/ml) for at least 3 weeks.

Cells stably transfected with the pAC-y1sgRNA-cas9 plasmid targeting the yellow locus were used as Cas9 control cells (CTRL). Western blot analysis indicates that the kinetic of dTIS11 degradation in response to hypoxic culture condition is similar in this yellow KO cell line as compared to wild-type S2 cells (not shown).

For pMT constructs, transcription driven by Metallothionein promoter was induced by treating the cells with CuSO4 (0.5 mM) for the indicated time. For mRNA half-life measurements, transcription was blocked by actinomycin D (5 µg/ml) and the cells were harvested at the indicated time points. The one-phase decay (nonlinear regression) of the ImPL3/Rpl32 mRNA ratio was used for calculation of ImpL3 mRNA half-life (Y0 = 100, plateau = 0) as Rpl32 mRNA level was previously reported to be marginally affected by actinomycin D treatment of S2 cells^22^.

### Plasmids

The luciferase reporter genes were described previously^22^. Wild-type 3′UTR of *ImpL3* gene was amplified by RT-PCR with the oligonucleotides FOR: 5′-aattggatccatcaaagcattcaactaacag-3′/REV: 5′-aattgtcgacaaacttaaaatttaaacagtttattc-3′. The DNA fragments were inserted between BamHI and SalI sites of the pMT-luciferase vector to generate a Luc-3′UTR IMPL3 reporter gene. A Luc-IMPL3ΔARE reporter gene was generated by cloning a mutated 3′UTR of ImpL3 obtained by a 2-step PCR-based mutagenic procedure. The sequence was verified by sequencing. GFP and dTIS11 expressing plasmids were described previously^22,24^.

The dTIS11 Cas9 plasmid was generated as described previously^42^ using the oligo nucleotides FOR: 5′-ttcggacgaggcccgcgcccaac-3′/REV: 5′-aacgttgggcgcgggcctcgtcc-3′.

Plasmids for *in vitro* transcription of wild-type ImpL3 and scrambled ImpL3 3′UTR were generated by hybridization of 2 primers followed by cloning into pBlueScript between the BamHI and EcoRI sites. The primers used were:

5′aattcgtttatttttagtccacctctcaatttatttattatttttatgaattagg3′/

5′gatccctaattcataaaaataataaataaattgagaggtggactaaaaataaacg3′ (ImpL3) and

5′aattcatttttttgtttttctgataattcgtgtatgtccttcacaaattgttaaatg3′

5′gatccatttaacaatttgtgaaggacatacacgaattatcagaaaaacaaaaaaatg3′ (scrambled ImpL3).

### Polysome fractionation

Cell lysis, ultracentrifugation and gradient collection was performed as in^73^.

### Genotyping of dTIS11 KO cell line

Cas9-mediated editing was verified by surveyor nuclease assay^74^ and genome mapping of the *dTis11* locus in dTIS11 KO cells was performed by PCR. Adjacent loci (*CK-alpha*, *SMR* and *Tomosyne*) were amplified by PCR to verify the specificity of deletion. Primers used were:

*dTis11*: 5′aggagccaaagggttaagga3′/5′ccattcgatgccaaatatcc3′;

*CK-alpha*: 5′caagctgtaccgcattctgag3′/5′ctgcttcagcattgtccagtc3′;

*SMR*: 5′gcgataagaatgcagcaacagc3′/5′caccggaattcagttcgaatcg3′;

*Tomosyne*: 5′cgctccatggccagctggtgc3′/5′catggattgtacatggcatcgcccac3′.

Cells stably transfected with the pAC-y1sgRNA-cas9 plasmid (targeting the *Yellow* locus) were used as Cas9 control cells (CTRL).

### RNA sequencing

Library preparation, RNA sequencing and bioinformatic analyses of differential Gene Expression were performed at the BRIGHTcore platform (Brussels, Belgium). RNA sequencing was performed on biological triplicates of CTRL and dTIS11 KO S2 cells grown in normoxia (21% O_2_), in hypoxia (1% O_2_ for 18 h) and upon recovery after hypoxia (90 min at 21% O_2_ after 18 h at 1% O_2_). 1 µg of each sample was used according to the standard sequencing protocol of Illumina (TrueSeq stranded mRNA). Quality control steps such as running the samples on a Bioanalyzer after library preparation were performed. The libraries were then sequenced on an Illumina HiSeq with a 150-base paired-end run. An average of 20 to 27 million reads was generated per sample. Read quality was determined using FastQC. The raw fasta files were deposited on the Sequence Read Archive (SRA) database under BioProject number: 373826.

### Bioinformatic analyses

Sequence reads were aligned to the *D*. *melanogaster* genome (BDGP6) using STAR and counted using HTseq. Finally, differential gene expression analysis was done using the edgeR method on the Degust platform (http://vicbioinformatics.com/degust/index.html), with FDR < 0.05 and fold change cut-off as indicated. Further statistical analyses and graphs were done using R (see Sup. methods).

AREScore analysis was performed according to^32^, with standard parameters and scoring options (http://AREScore.dkfz.de/AREScore.pl). To establish random lists of transcripts, we first defined a list of all genes expressed in S2 cells (minimal reads count of 10 in RNAseq of either normoxic or hypoxic S2 cells). We further randomly selected two independent groups of 900 genes among the 8780 genes detected as expressed in S2 cells by running the “sample” function of the R language (see Sup. methods). For each group of 900 genes, we extracted all described transcript isoforms from the RefSeq database to generate two lists of respectively, 2136 and 2175 transcripts.

### RNA analysis

Total RNA was purified using Tri reagent (MBI Fermentas) and analyzed by northern blot as in ref.^22^. RNA probe complementary to rp49 sequence (encoding ribosomal protein L32) was used to normalize RNA loading. Quantification of the radioactive signals was performed with a Phosphorimager (Molecular Dynamics). For qRT-PCR, cDNA was synthesized using the Prime ScriptTM RT Reagent Kit (Takara) according to manufacturer’s instructions. Quantitative PCR was performed on a StepOnePlus real-time PCR system (Applied Biosystems) using SYBR® Premix Ex TaqTM II (Takara) according to manufacturer’s instructions. Expression levels were normalized to Rpl32 (∆∆CT). The sequences of the primers used were: dTis11: 5′-acgaccaaaccatggaaact-3′/5′-ccttggcatatttgcgtttc-3′; ImpL3: 5′-caccgacatcctcaagaacat-3′/5′-gggattggacaccataagca-3′; Rpl32: 5′-gacgcttcaagggacagtatctg-3′/5′-aaacgcggttctg-catgag-3′. CecA1: 5′-gtcgctctcattctggccat-3′/5′gtcgctctcattctggccat-3′; Branchless: 5′-ccaaaccgcgggaatttctg- 3′/5′-cttcgtcttccccgctgata-3′.

### RNA Immunoprecipitation Assay

RIP experiments were performed essentially as described in ref.^75^. Immunoprecipitation was performed with a monoclonal anti-dTIS11 antibody^46^ or control murine IgG1.

### Purification of recombinant dTIS11 preparation and in vitro binding assays

dTIS11 protein was purified from BL21 DE3 bacteria transformed with a T7-dTIS11-6His plasmid. Briefly, bacteria were lysed after freezing for 20 min in lysis buffer (40 mM Hepes pH 7.8, 120 mM NaCl, 0.01% NP40, 0.1 mM EDTA, 5% glycerol, 10 mM 2-mercaptoethanol, 300 µg/ml lysozyme and Roche mini EDTA-free protease inhibitor cocktail) followed by sonication. After centrifugation, supernatant supplemented with imidazole (20 mM) was mixed with Ni-Sepharose beads. After washing, dTIS11-6His was eluted with elution buffer (300 mM imidazole, 40 mM Hepes pH 7.8, 500 mM NaCl, 0.01% NP40, 15% glycerol, 10 mM 2-mercaptoethanol, and protease inhibitors). The eluted fraction was desalted on PD-10 Columns and stored at −80 °C. Analysis of ImpL3 3′UTR-dTIS11 interaction by EMSA was performed as described in ref.^76^ with the following modifications. Purified recombinant His-tagged dTIS11 (1 µg) was incubated with labelled RNA probe (150.000 cpm) in Tris buffer (20 mM Tris pH 7.8, 3 mM MgCl_2_, 40 mM KCl, 2 mM DTT, 5% Glycerol) in the absence or the presence of increasing amounts of unlabeled RNA. Electrophoresis was performed on 5% polyacrylamide non-denaturing gels containing 6% glycerol at 7.5 mA for 16 hours at 4 °C.

ITC titrations were performed on an Affinity ITC (TA Instruments). All the measurements were done at 10 °C, titrating dTIS11 at a concentration of 108 μM into RNA fragments at a concentration of 10 μM to 15 μM. Prior to the measurements, dTIS11 and the different RNA fragments were dialyzed into the same buffer solution (20 mM MES pH 6.5, 300 mM NaCl, 5 µM ZnCl_2_, 0.5 mM DTT), degassed and equilibrated at the binding temperature. Sample concentrations were determined after dialysis or buffer exchange by absorbance measurements at 280 nm (dTIS11) or at 260 nm (RNA fragments). The ITC titrations were done at a stirring rate of 75 rpm with constant injection volumes of 2 μL of titrant into the cell (177 μL). All data were analyzed using NanoAnalyze and the final plots were generated with Origin. The RNA fragments used were: TNF ARE: 5′-cgauuuauuuauuuaga-3′ as previously described;^46^ CecA1: 5′-gauuauuuauaauuauuuauuuaaagaucuauuuauuc-3′; ImpL3 :5′-uguuuauuuuuaguccaccucucaauuuauuuauuauuuuuauga-3′; Scramble: 5′-auucuauuguuucuucuuauauauuguuauucuucacauaauguu-3′.

### Western blotting and antibodies

Cell lysis and western blot were performed as described previously^24^. Gel images were acquired on a Licor Odyssey FC imaging system using supersignal West pico (Thermo Scientific) chemiluminescent substrate.

### Dual Luciferase Assay

Dual-Luciferase® Reporter Assay from Promega was used after cell lysis according to manufacturer’s instruction.

### Measurement of S2 cell proliferation

Proliferation of CTRL and dTIS11 KO cells in recovery after 48 h hypoxia was assessed by flow cytometry counting (FACS Canto II, BD) using Polybead® Polystyrene 6.0 micron Microspheres (Polysciences). Briefly, cells were counted with Trypan blue and plated at 1.5 × 10^6^ cells/ml. 50 µl from a 200 times dilution of the Polybeads was added to 500 µl of cell suspension before flow cytometry analysis (FSC vs SSC). Number of cells/500 counted beads was recorded for each time point. Cells were counted at plating, after 48 h of hypoxia and every 24 h in normoxia recovery (up to 72 h). Cell proliferation index in recovery corresponds to the ratio of cell number at indicated time point/cell number at 48 h of hypoxia.

### JC-1 staining and detection

Mitochondrial polarization was assessed using MitoProbe^TM^ JC-1 Assay Kit (Life Technologies) according to manufacturer’s instructions. CTRL and dTIS11 KO cells were plated at 1.5 × 10^6^ cells/ml and cultivated 48 h in hypoxia. At the indicated time points, cells were harvested and washed in PBS. Cells were then suspended in 200 µl PBS with JC-1 dye (2 µM) and left at 25 °C for 20 min in the dark for staining. Cells were analyzed by flow cytometry after a final wash in PBS. One tube of cells was briefly incubated with 10 µM FCCP (Sigma-Aldrich) prior to staining with JC-1, as depolarization control. FACS data were analyzed using FlowJo® V10. Mitochondrial depolarization is indicated by a decrease in the red/green fluorescence intensity ratio. JC-1 index was calculated as MFI Red/MFI Green for live single cells and normalized to the JC-1 index for cells at 48 h hypoxia.

### Statistical analysis and graphs

Graphs were made using GraphPad Prism 5. The one-phase decay (nonlinear regression) was used for calculation of mRNA half-lifes (Y0 = 100, plateau = 0). Other statistical tests are as indicated in the text, p-values for multiple testing were corrected using Bonferroni’s correction method.

## Electronic supplementary material

Supplementary figures and methods

Supplementary tables
