# Supplementary material for: ARE-mediated decay controls gene expression and cellular metabolism upon oxygen variations
Source: Sci Rep. 2018 Mar 26;8:5211. doi: 10.1038/s41598-018-23551-8 (PMC5980108; doi:10.1038/s41598-018-23551-8)
Supplement: Supplementary file 1 — Supplementary figures and methods [file 41598_2018_23551_MOESM1_ESM.docx]

**ARE-mediated decay controls gene expression and cellular metabolism upon**

**oxygen variations**

Bérengère de Toeuf, Romuald Soin, Abdelkarim Nazih, Marija Dragojevic, Dukas Jurenas, Nadège Delacourt, Long Vo Ngoc, Abel Garcia-Pino, Véronique Kruys, and Cyril Gueydan.


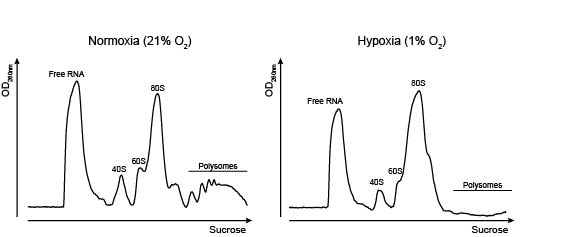
**Supplementary Figure S1**. **Hypoxia leads to a general down modulation of translation in S2 cells.** Polysome profiles of S2 cells grown at 1% O_2_ for 18h (right) or left at 21% O_2_ (left) for the same time period. After incubation, cells were treated with cycloheximide (100 µg/ml) and lysed. Cytoplasmic lysates were loaded onto 15-50% sucrose gradients and ultracentrifuged. Gradients were fractionated and O.D. 260nm was measured online with a Brandel type 11 optical unit.

**Supplementary Figure S2:**

**Full size image of the western blot experiment presented in figure 5A**

**
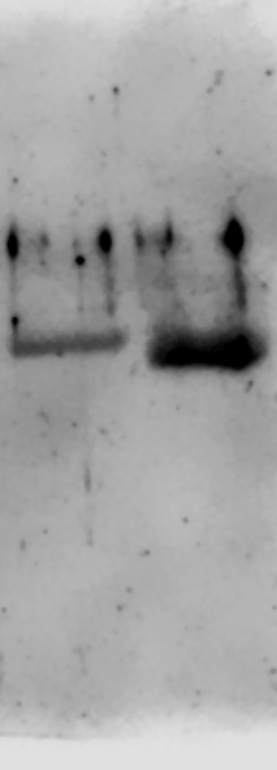
**

**Supplementary methods:** R scripts used in this study

### Generation of randomized controls for AREscore analysis in R

To generate a list of 900 random genes from genes expressed in S2 cells (8780 genes with one read count ≥ 10 in at least one RNAseq sample):

Random= S2[sample(1:nrow(S2), 900, replace=FALSE),]

To get all isoforms from those genes by Refseq number (NM_......), using BiomaRt:

Set the database:

ensembl=useMart("ensembl", dataset="dmelanogaster_gene_ensembl")

Get Refseq numbers:

getBM(attributes=c("refseq_mrna", "flybase_gene_id") , filters="flybase_gene_id", values=Random$GeneID, mart=ensembl)

### Statistical test of difference distributions using multiple Kolmogorov-Smirnov tests in R

For Kolmogorov-Smirnov test:

ks.test (1$Score, 2$Score) and store p-value in object named pval

For adjusted p-values for multiple testing:

p.adjust (pval, method=”bonferonni”)

### Chi-sqared test on multiple groups in R

Categorize AREscore in <2 or ≥2:

Transform (df, AREscore = ifelse (df$Score < 2, "low", "high"))

Create contingency table:

TABLE = table (df$group, df$AREscore)

Chi-squared test with post-hoc pairwise comparison (using library “fifer”):

chisq.post.hoc (TABLE, test ="chisq.test", control="bonferroni", digit=25)

**Supplementary Tables: (see attached Excel file)**

**Table S1**. List of transcripts upregulated in CTRL S2 cells upon hypoxia.

**Table S2.** List of transcripts downregulated in CTRL S2 cells upon hypoxia.

**Table S3.** List of AREScore ≥ 2 transcripts downregulated in CTRL and dTIS11 KO S2 cells upon reoxygenation (90 min.) after hypoxia (18h).

**Table S4.** List of AREScore < 2 transcripts downregulated in CTRL and dTIS11 KO S2 cells upon reoxygenation (90 min.) after hypoxia (18h).
